# Essential Oil of Common Sage (*Salvia officinalis* L.) from Jordan: Assessment of Safety in Mammalian Cells and Its Antifungal and Anti-Inflammatory Potential

**DOI:** 10.1155/2013/538940

**Published:** 2013-10-09

**Authors:** M. S. Abu-Darwish, C. Cabral, I. V. Ferreira, M. J. Gonçalves, C. Cavaleiro, M. T. Cruz, T. H. Al-bdour, L. Salgueiro

**Affiliations:** ^1^Department of Basic and Applied Sciences, Shouback University College, Al-Balqa Applied University, Al-Shouback 71911, Jordan; ^2^Center for Pharmaceutical Studies/Faculty of Pharmacy, Pólo das Ciências da Saúde, Azinhaga de Santa Comba, Universidade de Coimbra, 3000-548 Coimbra, Portugal; ^3^Centro de Neurociências e Biologia Celular, Universidade de Coimbra, 3000-295 Coimbra, Portugal; ^4^Faculdade de Farmácia, Universidade de Coimbra, 3000-295 Coimbra, Portugal; ^5^Department of Agricultural Sciences, Shouback University College, Al-Balqa Applied University, Al-Shouback 71911, Jordan; ^6^Laboratório de Farmacognosia, Faculdade de Farmácia da Universidade de Coimbra, Pólo das Ciências da Saúde, Azinhaga de Santa Comba, 3000-548 Coimbra, Portugal

## Abstract

*Salvia officinalis* L. (Lamiaceae) is a Mediterranean species, naturalized in many countries. In Jordan, it is used in traditional medicine as antiseptic, antiscabies, antisyphilitic, and anti-inflammatory, being frequently used against skin diseases. This study aimed the assessment of the antifungal and anti-inflammatory potential of its essential oils, and their cytotoxicity on macrophages and keratinocytes. The oils were investigated by gas chromatography and gas chromatography-mass spectrometry and the antifungal activity was evaluated against yeasts, dermatophyte and *Aspergillus* strains. Assessment of cell viability was made by the 3-(4,5-dimethylthiazol-2-yl)-2,5-diphenyltetrazolium bromide assay and the *in vitro* anti-inflammatory potential was evaluated by measuring nitric oxide production using lipopolysaccharide-stimulated mouse macrophages. The main compounds of *S. officinalis* oils were 1,8-cineole (39.5–50.3%) and camphor (8.8–25.0%). The oils revealed antifungal activity against dermatophyte strains and significantly inhibited NO production stimulated by LPS in macrophages, without affecting cell viability, in concentrations up to 0.64 **μ**L/mL. This is the first report addressing the *in vitro* anti-inflammatory potential of *S. officinalis* oil. These findings demonstrated that bioactive concentrations of *S. officinalis* oils do not affect mammalian macrophages and keratinocytes viability making them suitable to be incorporated in skin care formulations for cosmetic and pharmaceutical purposes.

## 1. Introduction

The genus *Salvia* L. (Lamiaceae) comprises about 900 species, spread throughout the world, some of which with great economic value since they are used as spices and flavoring agents by perfumery and cosmetic industries [1, 2].

*Salvia *sp. has also been used for a long time in folk medicine as medication against fever, rheumatism, perspiration, sexual debility, and in the treatment of chronic bronchitis, as well as mental and nervous diseases [3].

The most well-known species are common sage (*Salvia officinalis* L.), trilobed sage (*Salvia fruticosa* Mill.), and Spanish sage (*Salvia lavandulifolia* Vahl) [4]. 

*S. officinalis* (sage, garden sage, or common sage) is a perennial, evergreen subshrub, with woody stems, grayish leaves, and blue to purplish flowers. It is native to the Mediterranean region, being currently cultivated in various countries [4, 5]. Sage is one of the oldest medicinal plants, and the etymology of the Latin name suggests its healing properties, with *Salvia *deriving from the Latin verb *salvare = *to save or to cure, and *officinalis *also means medicinal. 

Sage is largely used as a savory food flavoring either as dried leaves or essential oil [6]. Sage leaves and its essential oil possess carminative, antispasmodic, antiseptic, astringent, and antihidrotic properties [4]. The predominant medicinally valuable metabolites are monoterpenes (e.g., *α*- and *β*-thujone, 1,8-cineole, camphor), diterpenes (e.g., carnosic acid) triterpenes (oleanoic and ursolic acids), and phenolic compounds like rosmarinic acid [7–9].

Essential oil of sage and their preparations are externally used for inflammations and infections of the mucous membranes of throat and mouth (stomatitis, gingivitis, and pharyngitis). Internally, the essential oil is used for dyspeptic symptoms and excessive perspiration [4].

*S. officinalis* produces monoterpenes with a broad range of carbon skeletons, including acyclic, monocyclic, and bicyclic compounds. Three distinct monoterpene synthases are responsible for the first steps in the formation of the most characteristic monoterpenes of *S. officinalis* essential oil. The (+)-sabinene synthase catalyzes the production of sabinene, which undergoes further rearrangements leading to the two major monoterpenes, *α*- and *β*-thujone. The 1,8-cineole synthase produces in one-step 1,8-cineole. Finally, (+)-bornyl diphosphate synthase produces bornyl diphosphate, which is subsequently hydrolyzed to borneol and then oxidized to camphor [10–15].

Environmental conditions such as temperature, day length, and light influence quantitative compositions of essential oils [16]. These conditions change significantly and predictably during the vegetation period, leading to a pattern of seasonal variation in plant metabolites, that is, generally repeated every year. In *Salvia*, the major monoterpenes 1,8-cineole, camphor, and the two thujones show pronounced dynamics during a vegetative cycle that has been confirmed under different geographical conditions [17–20]. According to Grausgruber-Gröger et al. 2012 [21], 1,8-cineole steadily decreases during a vegetative period (approx. May to October), camphor peaks in the middle of the vegetative period, and *α*- and *β*-thujone increase gradually during the vegetative period. 

The antibacterial properties of sage oils have been attributed to the presence of 1,8-cineole, thujone, and camphor [2]. However in 2007, Pinto et al. [22] stated that thujones do not play an important role against yeasts and filamentous fungi. This work also suggests 1,8-cineole and camphor as the main responsible for the antifungal activity in the tested strains.

Common sage is well known in Palestitian traditional medicine by possessing antimicrobial properties [23]. In 2008, a taxonomical and pharmacological survey of therapeutic plants in Jordan indicates the main traditional uses *S. officinalis* as antiseptic, antiscabies, antisyphilitic, and anti-inflammatory, being frequently used against skin and eye diseases and also in pleurisy [24, 25]. In Jordan and the Middle East, it is also being reported the use of common sage for fever, digestive disorders, and stomach ache [26]. 

Thus, taking into account these traditional uses of *S. officinalis*, in the present work we aim to characterize the chemical composition of the essential oil obtained from the aerial parts of *S. officinalis *collectedin different regions of Jordan, as well as to evaluate the antifungal and the anti-inflammatory activities of the oil at concentrations safe to mammalian cells. 

## 2. Materials and Methods

### 2.1. Plant Material

Aerial parts of *S. officinalis* were collected during April, in six localities of Jordan: S1-Shoubak (South Jordan); S2:Ma'an (South Jordan); S3:Tafillah (South Jordan); S4:Jerash (North Jordan); S5:Ajloun (North Jordan); and S6:Amman (Middle Jordan). Voucher specimens were deposited at the Herbarium of Shoubak University College-Al-Balqa Applied University, Jordan with the following references: Abu-Darwish 1_2010 (S1); Abu-Darwish 4_2010 (S2); Abu-Darwish 7_2010 (S3); Abu-Darwish 10_2010 (S4); Abu-Darwish 13_2010 (S5); and Abu-Darwish 16_2010 (S6).

All the six samples were used for essential oil isolation and the oils were characterized. Only the oils chemically more distinct (S1 and S2) were chosen for biological assays.

### 2.2. Essential Oil Isolation

The essential oil of the six samples was isolated by hydrodistillation for 3 h using a Clevenger-type apparatus, according to the procedure described in the European Pharmacopoeia [27].

### 2.3. Gas Chromatography (GC)

Analytical GC was carried out in a Hewlett-Packard 6890 (Agilent Technologies, Palo Alto, CA, USA) chromatograph with a HP GC ChemStation Rev. A.05.04 data handling system, equipped with a single injector and two flame ionization detectors (FIDs). A graphpak divider (Agilent Technologies, part no. 5021-7148) was used for simultaneous sampling to two Supelco (Supelco, Bellefonte, PA, USA) fused silica capillary columns with different stationary phases: SPB-1 (polydimethylsiloxane 30 m × 0.20 mm, film thickness 0.20 *μ*m) and SupelcoWax-10 (polyethyleneglycol 30 m × 0.20 mm, film thickness 0.20 *μ*m). The oven temperature was programmed from 70°C to 220°C at 3°C/min and then held isothermal (15 min); injector temperature: 250°C (injection mode: split 1/40); detectors temperature: 250°C; carrier gas: helium, adjusted to a linear velocity of 30 Cm·s^−1^.

### 2.4. Gas Chromatography-Mass Spectrometry (GC-MS)

GC-MS was carried out in a Hewlett-Packard 6890 gas chromatograph fitted with a HP1 fused silica column (polydimethylsiloxane 30 m × 0.25 mm, film thickness 0.25 *μ*m), interfaced with an Hewlett-Packard mass selective detector 5973 (Agilent Technologies) operated by HP Enhanced ChemStation software, version A.03.00. GC parameters as described above; interface temperature: 250°C; MS source temperature: 230°C; MS quadrupole temperature: 150°C; ionization energy: 70 eV; ionization current: 60 *μ*A; scan range: 35–350 units; scans·s^−1^: 4.51.

### 2.5. Qualitative and Quantitative Analyses

Components of the volatile oils were identified by their retention indices on both SPB-1 and SupelcoWax-10 columns, calculated by linear interpolation relative to retention times of C_8_–C_24_ of *n*-alkanes and compared with those of reference compounds included in CEF laboratory database or literature data [28], and by their mass spectra by matching with reference spectra from the CEF laboratory own spectral database, Wiley/NIST database or literature data [28–30]. Relative amounts of individual components were calculated based on GC raw data areas without FID response factor correction.

### 2.6. Antifungal Activity Evaluation

#### 2.6.1. Fungal Strains

The antifungal activity of the oils obtained from the aerial parts of *S. officinalis* collected in Shoubak, South Jordan (S1) and Ma'an, South Jordan (S2), was evaluated against yeasts and filamentous fungi strains (*Aspergillus* spp. and dermatophytes).

*(1) Yeasts*. Three American Type Culture Collection (ATCC) type strains (*Candida albicans *ATCC 10231, *C. parapsilosis *ATCC 90018, *C. tropicalis* ATCC 13803); one CECT (Colección Española de Cultivos Tipo) type strain (*Cryptococcus neoformans *CECT 1078); and two clinical strains are isolated from recurrent cases of vulvovaginal candidiasis (*Candida guillermondii* MAT23 and *C. krusei* H9).

*(2) Aspergillus Species*. Two ATCC type strains (*Aspergillus niger *ATCC 16404 and* A. fumigatus *ATCC 46645) and one clinical strain are isolated from bronchial secretions (*A. flavus *F44).

*(3) Dermatophytes*. Three dermatophyte clinical strains are isolated from nails and skin (*Epidermophyton floccosum* FF9*, Trichophyton mentagrophytes *FF7, and *Microsporum canis *FF1) and four CECT (Colección Española de Cultivos Tipo) type strains (*Trichophyton rubrum* CECT 2794*, M. gypseum *CECT 2908, and *T. mentagrophytes* var. *interdigitale *CECT 2958, *T. verrucosum* CECT 2992).

*Candida parapsilosis* ATCC 90018 was used as control.

The fungal isolates were identified by standard microbiology methods and stored on Sabouraud broth with glycerol at –70°C. Prior to antifungal susceptibility testing, each isolate was inoculated on Sabouraud agar to ensure optimal growth characteristics and purity.

#### 2.6.2. Antifungal Activity Methods

All chemicals were supplied by Sigma Aldrich, except fluconazole, supplied by Pfizer, UK under the name Diflucan.

Broth macrodilution methods based on the Clinical and Laboratory Standards Institute (CLSI) reference protocols M27-A3 [31] and M38-A2 [32], for yeasts and filamentous fungi, respectively, were used to determine minimum inhibitory concentrations (MICs) and minimum lethal concentrations (MLCs) of the essential oil.

The serial doubling dilution of the essential oil was prepared in dimethyl sulfoxide (DMSO), with concentrations ranging from 0.32 to 20 *μ*L/mL. Final concentration of DMSO never exceeded 2%. Recent cultures of each strain were used to prepare the cell suspension adjusted to 1-2 × 10^3^ cells per mL for yeasts and 1-2 × 10^4^ cells per mL for filamentous fungi. The concentration of cells was confirmed by viable count on Sabouraud agar. The test tubes were incubated aerobically at 35°C for 48 h/72 h (*Candida* spp. and *Aspergillus* spp./*Cryptococcus neoformans*) and at 30°C for 7 days (dermatophytes) and MICs were determined. To evaluate MLCs, aliquots (20 *μ*L) of broth were taken from each negative tube after MIC reading and cultured in Sabouraud dextrose agar plates. Plates were then incubated at 35°C for 48 h (*Candida* spp. and *Aspergillus* spp.) and 72 h for *Cryptococcus neoformans* and 30°C for 7 days (dermatophytes). In addition, two reference antifungal compounds, amphotericin B (Sigma Aldrich) and fluconazole (Pfizer), were used to control the sensitivity of the tested microorganisms. All tests were performed in Roswell Park Memorial Institute medium (RPMI 1640 medium). For each strain tested, both growth conditions and sterility of the medium were checked in two control tubes. The innocuity of DMSO was also checked at the highest tested concentration. All experiments were performed in triplicate and repeated if the results differed.

### 2.7. Anti-Inflammatory Evaluation

#### 2.7.1. Cell Culture and Materials

Raw 264.7 (ATCC number: TIB-71), a mouse macrophage cell line kindly supplied by Dr Otília Vieira (Centro de Neurociências e Biologia Celular, Universidade de Coimbra, Coimbra, Portugal), was cultured in Iscove's Modified Dulbecco's Eagle Medium supplemented with 10% noninactivated fetal bovine serum, 100 U/mL penicillin, and 100 **μ**g/mL streptomycin at 37°C in a humidified atmosphere of 95% air and 5% CO_2_.

#### 2.7.2. Nitric Oxide (NO) Measurement

The anti-inflammatory activity of the oils from samples S1 and S2 was evaluated in the mouse macrophage cell line Raw 264.7.

The production of NO was measured by the accumulation of nitrite in the culture supernatants, using a colorimetric reaction with the Griess reagent [33]. The cells were plated at 0.6 × 10^6^ cells/well in 48-well culture plates, allowed to stabilize for 12 h, and then incubated with culture medium (control), or stimulated with 1 *μ*g/mL LPS, or with 1 *μ*g/mL LPS in the presence of different concentrations of the essential oil during 24 h. Briefly, 170 *μ*L of culture supernatants was collected and diluted with equal volume of the Griess reagent (0.1% (w/v) N-(1-naphthyl) ethylenediamine dihydrochloride and 1% (w/v) sulphanilamide containing 5% (w/v) H_3_PO_4_) during 30 min, in the dark. The absorbance at 550 nm was measured using an automatic plate reader (SLT, Austria). Nitrite concentration was determined using a sodium nitrite standard curve.

### 2.8. Evaluation of Cytotoxicity

#### 2.8.1. Cell Culture and Materials

The human keratinocyte cell line HaCaT, obtained from DKFZ (Heidelberg), was kindly supplied by Dr Eugénia Carvalho (Centro de Neurociências e Biologia Celular, Universidade de Coimbra, Coimbra, Portugal). Keratinocytes were cultured in Dulbecco's Modified Eagle Medium (high glucose) supplemented with 4 mM glutamine, 10% heat inactivated fetal bovine serum, 100 U/mL penicillin, and 100 *μ*g/mL streptomycin, at 37°C in a humidified atmosphere of 95% air and 5% CO_2_. In the experiments, cells were monitored by microscope observation in order to detect any morphological change.

#### 2.8.2. MTT Assay for Cell Viability

Cell viability was assessed for the oils from samples S1 and S2 using the mouse macrophage cell line Raw 264.7 and the human keratinocyte cell line HaCaT.

Assessment of cell viability was made through a colorimetric assay, using MTT [34]. The HaCaT cells (0.2 × 10^6^ cells/well, cultured in 48-well microplates) were incubated in a final volume of 600 *μ*L, allowed to stabilize for 12 h, and then incubated for 24 h with different concentrations of the essential oil. After adding 60 *μ*L of MTT solution (5 mg/mL in PBS) to each well, the cells were further incubated at 37°C for 15 min, in a humidified atmosphere of 95% air/5% CO_2_. Supernatants were then discarded and 300 *μ*L of acidified isopropanol (0.04 N HCl in isopropanol) was added to the cultures and mixed thoroughly to dissolve the dark blue crystals of formazan. Formazan quantification was performed using an ELISA automatic microplate reader (SLT, Austria) at 570 nm, with a reference wavelength of 620 nm.

In the case of the cell line Raw 264.7, after collection of 170 *μ*L of culture supernatants for NO measurement, 43 *μ*L of MTT solution (5 mg/mL in PBS) was added and cells were incubated at 37°C for 15 min, in a humidified atmosphere of 95% air and 5% CO_2_. Supernatants were then discarded, and 300 *μ*L of acidified isopropanol (0.04 N HCl in isopropanol) was added to the cultures and mixed thoroughly to dissolve the dark blue crystals of formazan. Formazan quantification was performed as described above.

### 2.9. Data Analysis

All the experiments were performed in duplicate, being the results expressed as mean ± SEM of three independent experiments. The means were statistically compared using one-way ANOVA, with a Dunnett's multiple comparison test. The differences between the means were considered significant for values of *P* < 0.01. The statistical tests were applied using GraphPad Prism, version 5.02 (GraphPad Software, San Diego, CA, USA).

## 3. Results

### 3.1. Essential Oil Composition

The essential oil of the six samples was obtained in yields of 1.2–2.8% (w/w), based on the dry weight of the plant.

The qualitative and quantitative composition of the oils is presented in Table 1, where the compounds are listed by order of their elution on a polydimethylsiloxane column.

In total, 25 compounds were identified accounting for 92.7–99.0%. The oils did not show a significant difference in composition between them with the oxygen containing monoterpenes representing the major percentage (72.1–82.1%), with slight variations in the amounts of the main compounds: 1,8-cineole (39.5–50.3%) and camphor (8.8–25.0%). In these samples, thujones, characteristic compounds of sage oils, are present in very low amounts (*α*-thujone 1.2–3.3% and *β*-thujone 0.9–9.9%).

### 3.2. Antifungal Activity

Evaluation of MIC and MLC of the essential oil showed a variability of inhibition among all the fungal strains tested. The results of the antifungal tests are summarized in Table 2.

The dermatophyte strains showed more sensibility to these oils when compared with *Candida* and *Aspergillus* strains, particularly for *Trichophyton rubrum* and *Epidermophyton floccosum* with MIC of 0.64 *μ*L/mL. Among the tested yeasts, *Cryptococcus neoformans* was the strain that showed more sensibility with MIC of 0.64 *μ*L/mL.

### 3.3. Effect of the Essential Oil on Nitric Oxide Production Induced by LPS

**Figure 1:**
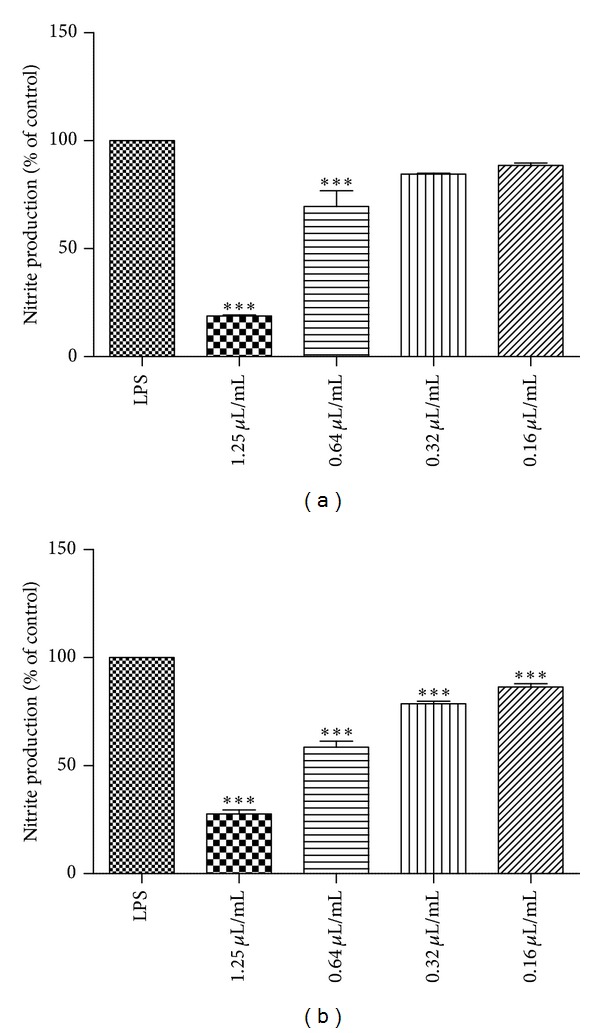
Effect of essential oil of *Salvia officinalis* aerial parts on NO production in macrophages; (a) S1 (*S. officinalis *collected in Shoubak, South Jordan) and (b) S2 (*S. officinalis *collected in Ma'an, South Jordan). Results are expressed as a percentage of nitrite production by control cells maintained in culture medium. Each value represents the mean ± SE From 3 experiments, performed in duplicate (***P < 0.01, compared to LPS).

The effect of essential oil on NO production triggered by LPS was evaluated in the mouse macrophage cell line Raw 264.7. After macrophages stimulation with LPS in the presence of the four concentrations of the oil, nitrite production induced by LPS was reduced to 18.81% ± 0.48 (1.25 *μ*L/mL), 69.60% ± 7.16 (0.64 *μ*L/mL), 84.60% ± 0.34 (0.32 *μ*L/mL), and 88.58% ± 1.11 (0.16 *μ*L/mL) for S1 (Figure 1(a)) and to 27.74% ± 1.88 (1.25 *μ*L/mL), 58.53% ± 2.72 (0.64 *μ*L/mL), 78.68% ± 1.19 (0.32 *μ*L/mL), and 86.46% ± 1.52 (0.16 *μ*L/mL) for S2 (Figure 1(b)).

### 3.4. Evaluation of Cell Viability

#### 3.4.1. Effect of the Essential Oil on Macrophages Viability

**Figure 2:**
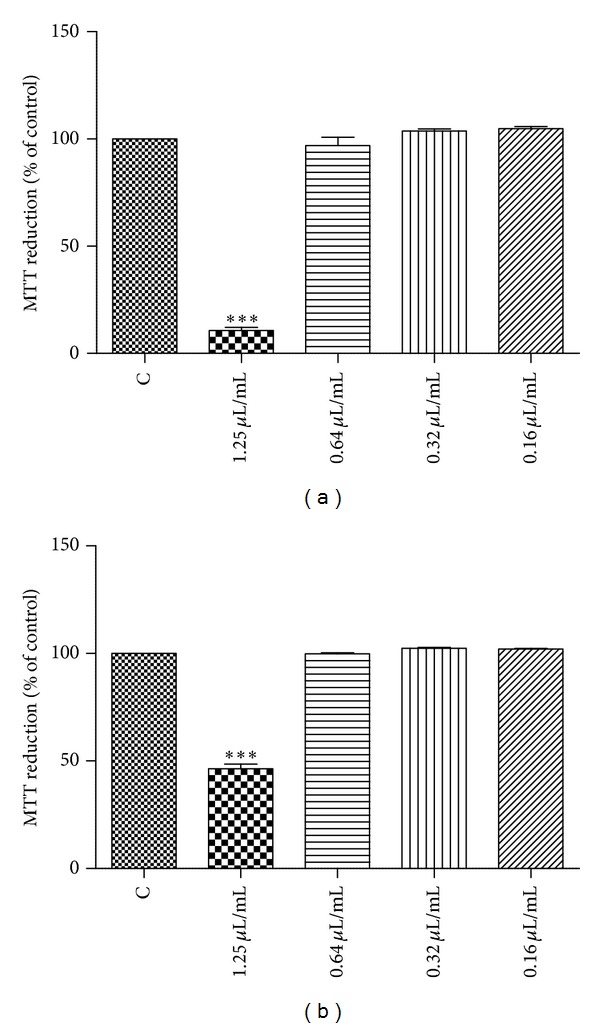
Effect of essential oil of *Salvia officinalis* aerial parts on macrophages viability (MTT assay); (a) S1 (*S. officinalis *collected in Shoubak, South Jordan) and (b) S2 (*S. officinalis *collected in Ma'an, South Jordan). Results are expressed as a percentage of MTT reduction by control cells maintained in culture medium. Each value represents the mean ± SEM from three experiments, performed in duplicate (***P < 0.01, compared to control).

To evaluate the possible cytotoxic activity of the oil in different mammalian cell types, the MTT assay was used both in macrophages and keratinocytes. Twenty-four hours of LPS exposure had no significant effect on macrophages viability. As shown in Figure 2(a), 1.25 *μ*L/mL of S1 decreased MTT reduction to 10.69% ± 1.44, while for concentrations of 0.64, 0.32, and 0.16 *μ*L/mL, the oil did not show significant cytotoxicity in macrophages, with values of 96.97% ± 3.88, 103.70% ± 0.96 and 104.9% ± 0.92, respectively. For S2, in the concentration of 1.25 *μ*L/mL, the MTT reduction decreased to 46.50% ± 2.12, while for concentrations of 0.64, 0.32, and 0.16 *μ*L/mL, the oil did not show significant cytotoxicity in macrophages, with values of 99.85% ± 0.44, 102.40% ± 0.36 and 102.0% ± 0.29, respectively, (Figure 2(b)).

#### 3.4.2. Effect of the Essential Oil on Keratinocytes Viability

**Figure 3:**
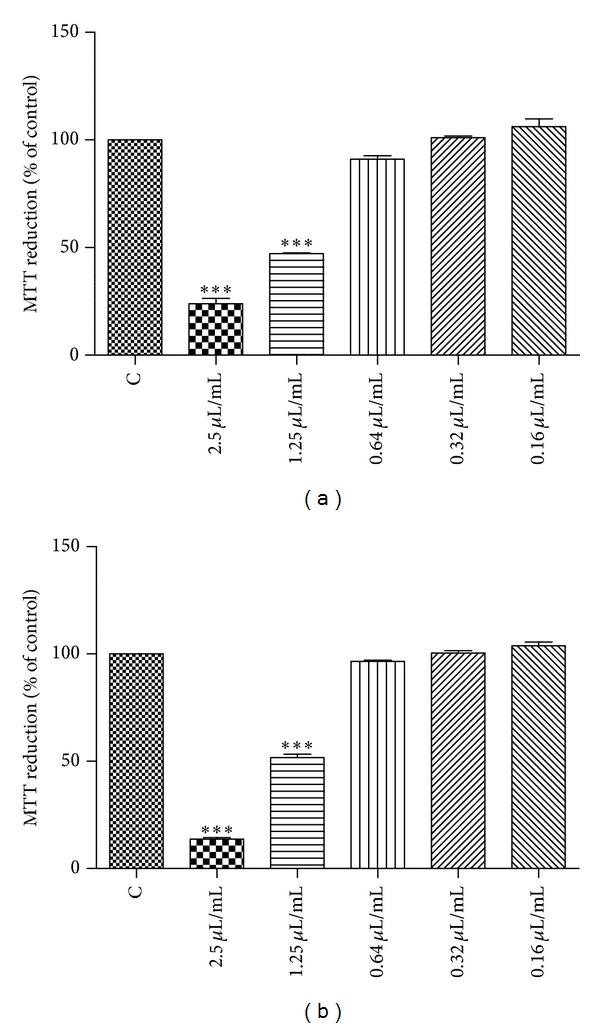
Effect of essential oil of *Salvia officinalis *aerial parts on keratinocytes viability (MTT assay); (a) S1 (*S. officinalis *collected in Shoubak, South Jordan) and (b) S2 (*S. officinalis *collected in Ma'an, South Jordan). Results are expressed as a percentage of MTT reduction by control cells maintained in culture medium. Each value represents the mean ± SEM from three experiments, performed in duplicate (***P < 0.01, compared to control).

As shown in Figures 3(a) and 3(b), 24 h of cells incubation with 2.5 and 1.25 *μ*L/mL of S1 and S2 essential oil decreased the MTT reduction by HaCaT in comparison with the control values to 23.93% ± 2.45 and 13.71% ± 0.71 (2.5 *μ*L/mL), 47.25% ± 0.30, and 51.66% ± 1.66 (1.25 *μ*L/mL), respectively. However, incubation of HaCaT with 0.64, 0.32 and 0.16 *μ*L/mL of the oil, during 24 h, showed no cytotoxic effect, with values of 91.01% ± 1.62 and 96.55% ± 0.54 (0.64 *μ*L/mL), 101.10% ± 0.79, and 100.50% ± 1.10 (0.32 *μ*L/mL), 106.20% ± 3.58, and 103.80% ± 1.70 (0.32 *μ*L/mL).

A cell-free control was performed in order to exclude nonspecific effects of the oils on MTT (data not shown).

## 4. Discussion and Conclusions

The essential oil composition of *S. officinalis* is highly influenced by genetic and environmental factors, organ age, climate conditions, and seasonality [35]. Due to such variation, the sage essential oil composition sometimes does not match the profile defined by the standard ISO 9909 [36] described as follows: *α*-thujone (18–43%), *β*-thujone (3–8.5%), camphor (4.5–24.5%), 1,8-cineole (5.5–13%), humulene (0–12%), *α*-pinene (1–6.5%), camphene (1.5–7%), limonene (0.5–3%), linalool (free and esterified (1% maximum)), and bornyl acetate (2.5% maximum). 

The essential oil of the Jordan samples (S1–S6) does not show a significant difference in composition between them and match these intervals for the majority of the compounds; however, the relative amount for *α*-thujone is much lower (1.2–3.7%) and for 1,8-cineole (39.5–50.3%) is much higher.

These results are in agreement with the revision of Grausgruber-Gröger et al. 2012 [21], where they stated that 1,8-cineole steadily decreases during a vegetative period (approx. May to October), camphor peaks in the middle of the vegetative period, and *α*- and *β*-thujones increase gradually during the vegetative period. Our samples were collected in April, and they possess very low amount of thujones and a quite high amount of 1,8-cineole.

In 1990, Tucker et al. [37] categorized sage commercial oils in five chemotypes according to the amount of the major compounds: (i) camphor > *α*-thujone > 1,8-cineole > *β*-thujone; (ii) camphor > *α*-thujone > *β*-thujone > 1,8-cineole; (iii) *β*-thujone > camphor > 1,8-cineole > *α*-thujone; (iv) 1,8-cineole > camphor > *α*-thujone > *β*-thujone; and (v) *α*-thujone > camphor > *β*-thujone > 1,8-cineole. These samples from Jordan belong to the fourth group, with 1,8-cineole (39.5–50.3%) > camphor (8.8–25.0%) > *α*-thujone (1.2–3.7%) > *β*-thujone (0.1–3.1%, with the exception of S6 with 9.9%). This chemotype is not very usual.

Thujones have been reported to be toxic to both brain and liver cells, presenting *α*-thujone higher toxicity than *β*-thujone [38, 39]. The samples from Jordan are very promising as they possess very low amount of thujones, thereby evidencing its safety profile. Accordingly, the oils did not affect macrophages and keratinocytes viability at concentrations up to 0.64 *μ*L/mL. The absence of keratinocyte toxicity is crucial to make an essential oil suitable for inclusion in topical formulations. Indeed, there is a lack of knowledge addressing keratinocytes cytotoxicity induced by essential oils and a current screening in our lab points that keratinocytes are very sensitive to several essential oils, which highlights the use of safe bioactive concentrations of *S. officinalis *essential oil for topical applications.

Macrophages produced a variety of proinflammatory mediators, such as nitric oxide, upon activation with the pro-inflammatory stimulus LPS. Since NO is a well-established inflammatory marker, the inhibition of its production might be a useful strategy to treat inflammatoryrelated diseases. Therefore, we also evaluated, for the first time, the *in vitro* anti-inflammatory activity of *S. officinalis *essential oil. The oils significantly inhibited NO production elicited by LPS in macrophages which demonstrates its strong anti-inflammatory potential. However, sample 2 (S2) has a slightly higher activity than sample 1 (S1), what can be related to the chemical composition. Although the samples possess similar chemical composition, they are distinguished by the content of camphor. Moreover, the higher amount of camphor in S2 did not affect the cell viability.

Concerning the antifungal properties of the essential oil, the results presented here demonstrate that the dermatophyte strains showed more susceptibility to these oils when compared with *Candida* and *Aspergillus* strains, particularly for *Trichophyton rubrum* and *Epidermophyton floccosum* with MIC of 0.64 *μ*L/mL. Among the tested yeasts, *Cryptococcus neoformans* was the strain that showed more susceptibility with MIC of 0.64 *μ*L/mL.

Although the MIC and MLC results varied among tested organisms, in various cases the MIC was equivalent to the MLC, indicating fungicidal activity of the oils (Table 2). 

Taking together, our results demonstrated that bioactive concentrations of *S. officinalis *oils do not affect mammalian macrophages and keratinocytes viability making them suitable to be incorporated in skin care formulations for cosmetic and pharmaceutical purposes.

## Figures and Tables

**Table 1:** Chemical composition of *Salvia officinalis* essential oil from Jordan.

RI^a^	RI^p^	Compounds*	Percentage in samples (%)					
			S1	S2	S3	S4	S5	S6
922	1030	*α*-Thujene	0.2	0.1	0.1	0.1	1.8	0.1
930	1030	*α*-Pinene	3.0	2.6	4.8	2.0	2.2	2.5
943	1073	Camphene	3.2	3.9	3.1	2.4	0.2	2.7
970	1118	*β*-Pinene	7.3	4.1	3.3	3.9	3.4	4.2
980	1161	Myrcene	1.9	1.5	1.9	1.2	0.9	1.2
1011	1275	*p*-Cymene	0.3	0.2	0.6	0.2	0.5	0.2
1017	1213	1,8-Cineole	48.5	39.5	50.3	44.9	46.1	43.7
1054	1456	Camphenilone	0.6	0.2	0.1	0.1	0.4	0.2
1085	1425	*cis*-Thujone	2.6	1.2	1.5	3.7	1.5	3.3
1096	1442	*trans*-Thujone	3.1	0.9	0.1	1.5	9.9	2.2
1118	1515	Camphor	8.8	25.0	10.3	19.9	11.4	16.2
1144	1695	Borneol	2.2	1.7	2.4	2.1	1.5	1.7
1147		Isoborneol	1.0	0.7	0.1	1.4	1.1	0.7
1165	1622	Myrtenal	0.6	0.8	0.6	0.8	0.3	0.9
1169	1692	*α*-Terpineol	3.0	2.8	3.1	2.9	2.7	2.6
1241	1550	Linalyl acetate	0.3	0.1	0.3	0.8	0.4	0.7
1264	1574	Bornyl acetate	0.8	1.3	0.7	1.0	0.5	0.2
1268	2178	Thymol	0.4	0.1	0.1	t	0.1	0.1
1277	2206	Carvacrol	0.6	0.1	0.1	0.1	0.1	0.1
1330	1687	*α*-Terpenyl acetate	2.0	1.4	2.4	2.9	2.7	2.5
1408	1590	*E*-*β*-Caryophyllene	3.5	2.4	5.2	2.2	1.4	5.5
1442	1662	*α*-Humulene	1.0	1.0	1.4	0.7	0.4	2.5
1554	2111	Spathulenol	0.4	t	0.2	0.1	0.1	0.2
1557	1968	Caryophyllene oxide	0.8	0.6	1.0	1.3	0.2	0.9
1570	2070	Viridiflorol	2.9	0.5	1.7	1.1	0.9	0.5
		Monoterpene hydrocarbons	15.9	12.4	13.8	9.8	9.0	10.9
		Oxygen containing monoterpenes	74.5	75.8	72.1	82.1	78.7	75.1
		Sesquiterpene hydrocarbons	4.5	3.4	6.6	2.9	1.8	8.0
		Oxygen containing sesquiterpenes	4.1	1.1	2.9	2.5	1.2	1.6

		Total identified	99.0	92.7	95.4	97.3	90.7	95.6

*Compounds listed in order to their elution on the SPB-1 column.

t: traces (<0.05%).

RI^a^: Retention indices on the SPB-1 column relative to C_8_ to C_24_ *n*-alkanes.

RI^p^: Retention indices on the SupelcoWax-10 column relative to C_8_ to C_24_ *n*-alkanes.

**Table 2:** Antifungal activity (MIC and MLC) of *Salvia officinalis* essential oil from Jordan against *Candida*, dermatophyte, and *Aspergillus* strains.

Strains	S1		S2		Fluconazole		Amphotericin B	
	MIC^a^	MLC^a^	MIC^a^	MLC^a^	MIC^b^	MLC^b^	MIC^b^	MLC^b^
*Candida albicans *ATCC 10231	2.5	5	2.5	5	1	>128	N.T.	N.T.
*C. tropicalis *ATCC 13803	5	5	5	5	4	>128	N.T.	N.T.
*C. krusei *H9	5	5	2.5	5	64	64–128	N.T.	N.T.
*C. guillermondii* MAT23	2.5	2.5	1.25–2.5	1.25–2.5	8	8	N.T.	N.T.
*C. parapsilosis *ATCC 90018	5	10	5	5	<1	<1	N.T.	N.T.
*Cryptococcus neoformans *CECT 1078	1.25	1.25	0.64–1.25	1.25–2.5	16	128	N.T.	N.T.
*Epidermophyton floccosum *FF9	0.64	0.64–1.25	0.64–1.25	0.64–1.25	16	16	N.T.	N.T.
*Microsporum canis *FF1	1.25	2.5	1.25	1.25	128	128	N.T.	N.T.
*M. gypseum *CECT 2905	1.25–2.5	1.25–2.5	1.25	2.5	128	>128	N.T.	N.T.
*Trichophyton mentagrophytes* FF7	1.25	1.25	1.25	1.25	16–32	32–64	N.T.	N.T.
*T. mentagrophytes* var. *interdigitale* CECT 2958	1.25	2.5	1.25	2.5	128	≥128	N.T.	N.T.
*T. rubrum *CECT 2794	0.64	1.25	0.64	0.64–1.25	16	64	N.T.	N.T.
*T. verrucosum* CECT 2992	2.5	2.5	1.25–2.5	2.5	>128	>128	N.T.	N.T.
*Aspergillus niger* ATCC 16404	5	>20	5	>10	N.T.	N.T.	2	8
*A. fumigatus *ATCC 46645	2.5–5	>20	5	>10	N.T.	N.T.	2	4
*A. flavus *F44	10	>20	5	>10	N.T.	N.T.	1-2	4

Results were obtained from 3 independent experiments performed in duplicate.

^a^MIC and MLC were determined by a macrodilution method and expressed in *μ*L/mL (V/V).

^b^MIC and MLC were determined by a macrodilution method and expressed in *μ*g/mL (W/V).

N.T.: not tested.
